# High-Quality Draft Genome Sequence of *Kallotenue papyrolyticum* JKG1^T^ Reveals Broad Heterotrophic Capacity Focused on Carbohydrate and Amino Acid Metabolism

**DOI:** 10.1128/genomeA.01410-15

**Published:** 2015-12-03

**Authors:** Brian P. Hedlund, Senthil K. Murugapiran, Marcel Huntemann, Alicia Clum, Manoj Pillay, Krishnaveni Palaniappan, Neha Varghese, Natalia Mikhailova, Dimitrios Stamatis, T. B. K. Reddy, Chew Yee Ngan, Chris Daum, Kecia Duffy, Nicole Shapiro, Victor Markowitz, Natalia Ivanova, Nikos Kyrpides, Amanda J. Williams, Jessica K. Cole, Jeremy A. Dodsworth, Tanja Woyke

**Affiliations:** a School of Life Sciences, University of Nevada, Las Vegas, Las Vegas, Nevada, USA; b Nevada Institute for Personalized Medicine, University of Nevada, Las Vegas, Las Vegas, Nevada, USA; c Department of Energy Joint Genome Institute, Walnut Creek, California, USA; d SWCA Environmental Consultants, Las Vegas, Nevada, USA; e Department of Energy Pacific Northwest National Laboratory, Richland, Washington, USA; f Department of Biology, California State University San Bernardino, San Bernardino, California, USA

## Abstract

The draft genome of *Kallotenue papyrolyticum* JKG1^T^, a member of the order *Kallotenuales*, class *Chloroflexia*, consists of 4,475,263 bp in 4 contigs and encodes 4,010 predicted genes, 49 tRNA-encoding genes, and 3 rRNA operons. The genome is consistent with a heterotrophic lifestyle including catabolism of polysaccharides and amino acids.

## GENOME ANNOUNCEMENT

Strain JKG1^T^ was isolated from a cellulolytic enrichment in Great Boiling Spring, Nevada (1), using optical tweezers and a microfluidic device due to its capacity to grow aerobically on filter paper (optimum 55 °C) (2). Strain JKG1^T^ was described as a member of a new genus and species, *Kallotenue papyrolyticum*, and a new order, *Kallotenuales*, of the phylum *Chloroflexi* (2). It has broad heterotrophic activity, including the capacity to hydrolyze polysaccharides such as carboxymethylcellulose, microcrystalline cellulose, filter paper, xylan, and starch, and proteinaceous substrates such as casamino acids, tryptone, and peptone. *K. papyrolyticum* is one of several, new high-level taxonomic groups of thermophilic *Chloroflexi* isolated from Great Boiling spring (3).

The draft genome of strain JKG1^T^ was generated at the DOE Joint genome Institute (JGI) using Pacific Biosciences (PacBio) technology. A PacBio SMRTbell library was constructed and sequenced on the PacBio RS platform, which generated 304,235 filtered subreads totaling 854.0 Mbp. All general aspects of library construction and sequencing performed at the JGI can be found at http://www.jgi.doe.gov. The raw reads were assembled using HGAP (version 2.0.0) (4). The genome was annotated using Prodigal version 2.5 (5), as part of the JGI microbial annotation pipeline (6). The *K. papyrolyticum* draft genome is 4,475,263 bp in 4 contigs, encoding 4,010 predicted genes, including 49 tRNA-encoding genes, and three rRNA operons. The genome encodes enzymes for complete glycolysis, tricarboxylic acid cycle, and pentose phosphate pathways and dedicates a large amount of its genome to carbohydrate (10.1%; clusters of orthologous groups [COG] category G) and amino acid (8.9%; COG category E) metabolism. Analysis of the genome for carbohydrate-active enzymes (CAZymes) (7) revealed 171 total CAZymes, 55 of which are glycoside hydrolases (GHs), including GHs putatively involved in chitin (GH18), woody plant (GH53), and cellulose (GH5,6,9,10,51) depolymerization. This number of CAZymes and GH domains is similar to other cellulolytic thermophiles, such as *Thermotoga maritima* (8), *Caldicellulosiruptor* species (9), and *Dictyoglomus turgidum* (10).

Catabolism of organic compounds can be coupled to aerobic respiration. Similar to *Chloroflexus* (11), strain JKG1^T^ has a prototypical respiratory complex I and II to transfer electrons into the quinone pool; however, quinols are likely oxidized by an alternative complex III (ACIII) (12) rather than a *bc*_1_/*b*_6_*f* complex The presence of lactate and ethanol dehydrogenases, acetate-CoA ligase, and an iron-only hydrogenase suggest capacity for fermentation, however, fermentation of glucose, casamino acids, or yeast extract was not observed in JKG1^T^ cultures (2). Strain JKG1^T^ may be able to oxidize carbon monoxide through a putative type II carbon monoxide dehydrogenase (CODH) encoded by a cox gene cluster, although the functions of type II CODH are not firmly established (13). Known pathways for phototrophy, autotrophy, and anaerobic respiration are absent, although putative molybdopterin oxidoreductases and multicopper oxidases may be involved in anaerobic respiration.

PFAM domains representing proteins involved in outer membrane biogenesis and transport are not present, suggesting JKG1^T^ has a monoderm cell envelope structure similar to other *Chloroflexi* (14).

### Nucleotide sequence accession numbers.

This whole-genome shotgun project has been deposited in GenBank under accession numbers JAGA01000001 to JAGA01000004.
